# Racial diversity amongst Otolaryngology-Head and Neck Surgery programs in Canada

**DOI:** 10.1186/s40463-023-00650-9

**Published:** 2023-07-19

**Authors:** Garret Horton, Elysia Grose, Tanya Chen, Oluwaseun Daniel Davies, Dongho Shin, Ian Witterick, Paolo Campisi, Yvonne Chan

**Affiliations:** 1 Department of Otolaryngology-Head and Neck Surgery, University of Toronto, Toronto, Canada; 2 Department of Radiation Oncology, University of Calgary, Calgary, Canada; 3 Department of Otolaryngology-Head and Neck Surgery, Mount Sinai Hospital, University of Toronto, Toronto, Canada; 4 Department of Otolaryngology-Head and Neck Surgery, SickKids Hospital, University of Toronto, Toronto, Canada; 5 Department of Otolaryngology-H and N Surgery, St. Michael’s Hospital, 30 Bond Street, #8-163 CC North, Toronto, ON M5B 1W8 Canada

**Keywords:** Diversity, Gender, Equity, Inclusion

## Abstract

**Background:**

The Canadian landscape of racial diversity in academic OHNS programs is currently unknown, as to date Canadian medical organizing bodies have refrained from collecting race-based data. However, new policy guidelines by the Canadian Medical Association support the collection of data that may be used to support equity, diversity and inclusion programs. This study aims to describe the representation of visible minorities amongst academic OHNS departments and divisions in Canada at various levels of academic seniority.

**Methods:**

An online survey was distributed to members of the 13 academic OHNS department in Canada in 2022. The survey collected demographic data as well as each participant’s self-reported race and gender. The primary outcome was the comparison of the racial demographics of Canadian academic OHNS programs to Canadian census data. Secondary outcome measures assessed how demographics varied based on academic position and gender. Simple descriptive statistics were tabulated for all demographic variables. Chi-square goodness of fit analysis was used to compare survey results to anticipated demographics based on 2016 Canadian census data.

**Results:**

Of 545 surveys distributed, 224 surveys were completed (response rate of 41%); 67.9% or respondents were male and 32.1% were female. Of these respondents, 71 were residents, 26 lecturers, 54 assistant professors, 39 associate professors, and 34 full professors. There was significantly greater minority representation amongst residents (47.9%), assistant professors (39.6%), and lecturers (40.7%) compared to the Canadian population (25.3%) *p* < 0.001. Results also showed that there were significantly fewer female lecturers (25.9%, *p* = 0.01), assistant professors (31.5%, *p* = 0.006), and full professors (2.9%, *p* < 0.001) compared to an assumed even proportion of men and women in the population.

**Conclusions:**

Academic OHNS programs in Canada are more racially diverse than the Canadian population. However, women continue to be under-represented in more senior positions, especially women who are visible minorities. Further investigation into the systemic factors that may contribute to this disparity is needed as well as effective ways to promote diversity amongst academic OHNS departments at all levels of academic seniority.

**Graphical abstract:**

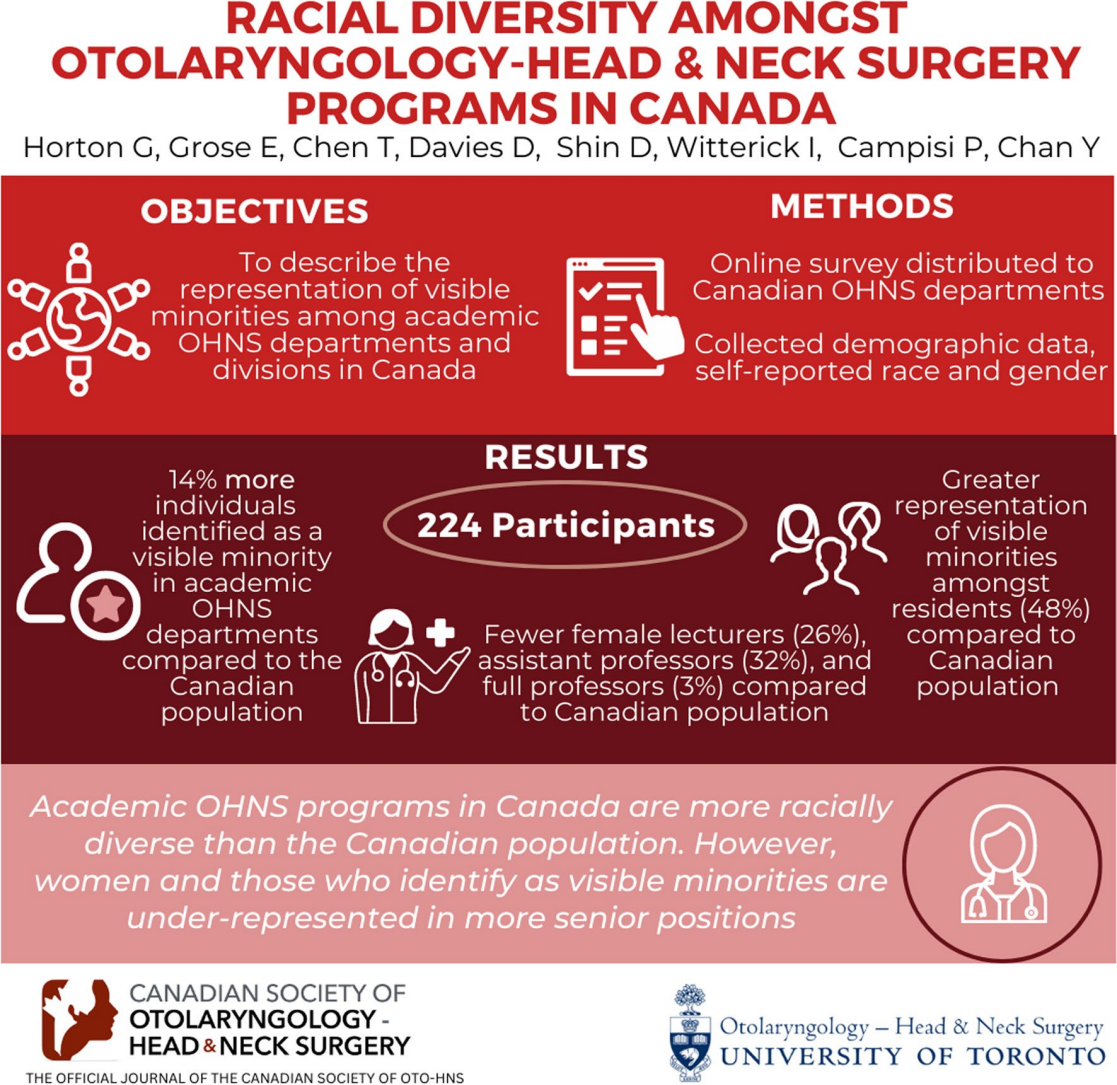

## Background

The benefits of increasing racial diversity in the academic physician population is well documented. A more diverse health care workforce has been attributed to decreased burn out and improved patient outcomes [1]. In academic centers, a racially heterogeneous population of physicians promotes biomedical research that ultimately addresses disparities in health access and outcomes in marginalized communities. In addition, minority patients are more likely to participate in clinical research studies when a member of their physician provider team is also a visible minority [2, 3]. Finally, increasing the ethnic or racial diversity of academic physicians provides greater opportunities for mentorship and training of the next cohort of diverse academic physicians [4].

In the US, Otolaryngology-Head and Neck Surgery (OHNS) is identified as a surgical subspecialty where racial disparities are particularly pronounced at all levels of training [1, 5–7]. When compared to other surgical specialties, OHNS had the lowest percentage (8.5%) of resident physicians designated by the Association of American Medical Colleges (AAMC) as individuals underrepresented in medicine (URM) [8]. The designation URM is defined by the AAMC as “those racial and ethnic populations that are underrepresented in the medical profession relative to their numbers in the general population.” In 2016, OHNS had the lowest percentage of African-American faculty compared to other surgical specialties. Asian Americans and Hispanic’s were underrepresented among faculty compared to otolaryngology residency programs as well [1].

The Canadian landscape of racial diversity of academic OHNS programs is currently unknown. Canadian medical organizing bodies have refrained from collecting race-based data which makes characterizing racial demographics challenging. However, new policy guidelines by the Canadian Medical Association support the collection of data that may be used to support equity, diversity and inclusion programs [9]. This study aims to provide a description of the representation of visible minorities amongst academic OHNS departments and divisions in Canada at various levels of academic seniority. The secondary aim is an assessment of gender equality at various academic levels.

## Methods

The study received Research Ethics Board approval from the University of Toronto Research Ethics Board (protocol # 00040425). An anonymous and voluntary online survey was distributed via email to staff Otolaryngology-Head and Neck surgeons and resident physicians among the 13 OHNS departments and divisions across Canada through email on January 30, 2022. Email addresses were obtained through program administrators. The survey was left active for one month and was hosted through Research Electronic Data Capture application (REDCap). The survey contained 5-items where individuals were asked to self-identify their ethnicity and gender. This survey collected anonymous information regarding each participant’s program location, academic rank, and whether they were based in an academic or community hospital (Table 1). The responses were collected anonymously. Data from the Canadian Census was used in this study to compare the proportion of visible minorities to that of the Canadian population [10].

**Table 1:** Survey distributed to OHNS programs nationwide

1. What is your academic title? (Select all that apply)
(a) Resident Physician (i.e. PGY1 – PGY5) (b) Fellow (c) Lecturer (d) Associate professor (e) Assistant professor (f) Professor
2. Where are you based?
(a) Community (b) Academic hospital based
3. Do you identify as transgender?
(a) Yes (b) No (c) Prefer not to answer
4. How would you describe your gender identity (select all that apply)?
(a) Agender (b) Genderqueer or genderfluid (c) Man (d) Muxe (e) Non-binary (f) Questioning or unsure (g) Two-spirit (h) Woman (i) Prefer not to disclose (j) Additional gender category/identity not listed (please specify below)
5. Regarding my ethnicity, how do you self-identify? Which of the following do you feel best describes you?
(a) Not a visible minority (“Caucasian in race or White in colour”) (b) Indigenous person – from Canada (c) Indigenous person – from another country (d) Black (e) Central Asian (Kazakh, Afghan, Tajik, Uzbek, Caucasus, etc.) (f) Chinese (g) Filipino (h) Japanese (i) Korean (j) Latina / Latino /Latinx / Hispanic (k) Middle Eastern (l) Southeast Asian (Cambodian, Indonesian, Laotian, Vietnamese, Thai, etc.) (m) South Asian (Indian, Pakistani, Sri Lankan, East Indian from Guyana, etc.)

Simple descriptive statistics (i.e. frequency) were tabulated for all demographic variables. Chi-square goodness of fit analysis was used to compare survey results to demographics that would be expected based on the 2016 Canadian census data [10]. A *p* < 0.05 was be considered statistically significant.

## Results

### Survey

In total, the survey was distributed to 545 individuals. There were 255 respondents out of the 545 people who were sent the survey (response rate of 41.3%), with one response being excluded as it was not completed in its entirety, for a total of 224.

Findings:

#### Gender diversity

**Fig. 1:**
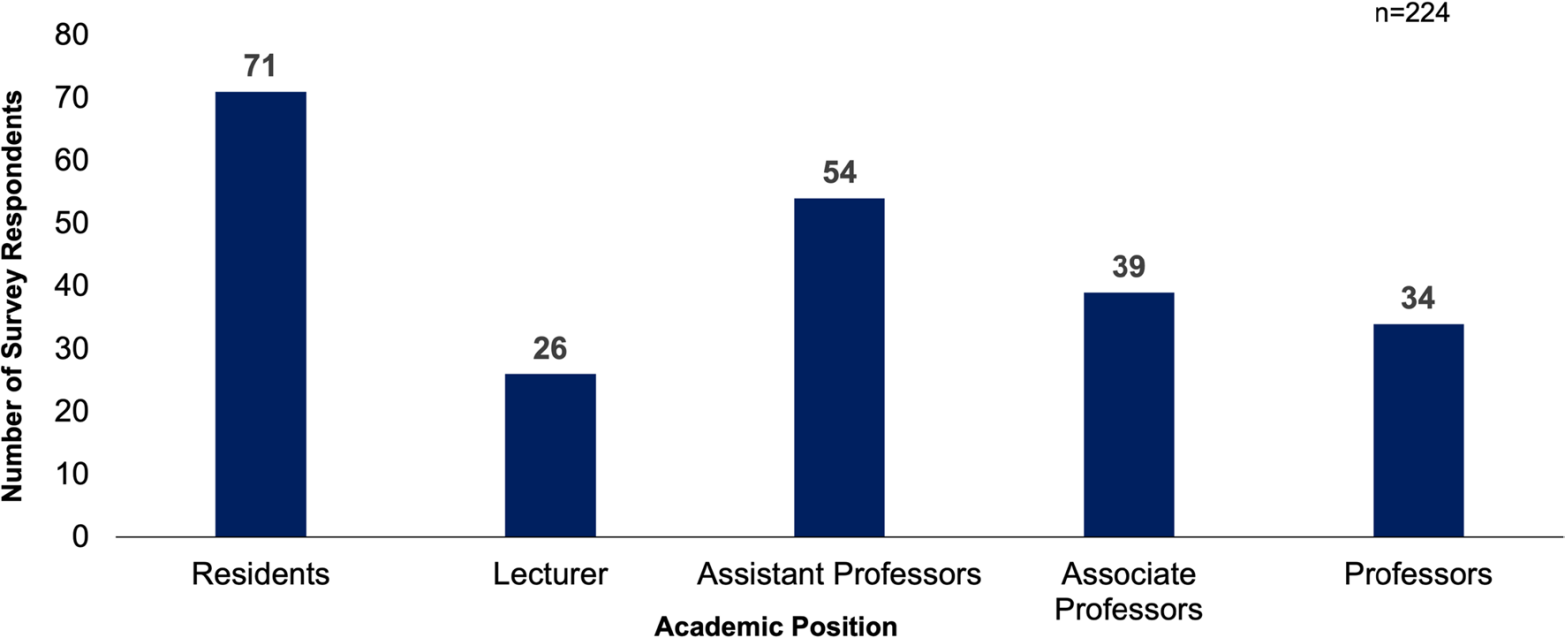
Number of survey respondents at each academic level (*n* = 224)

**Fig. 2:**
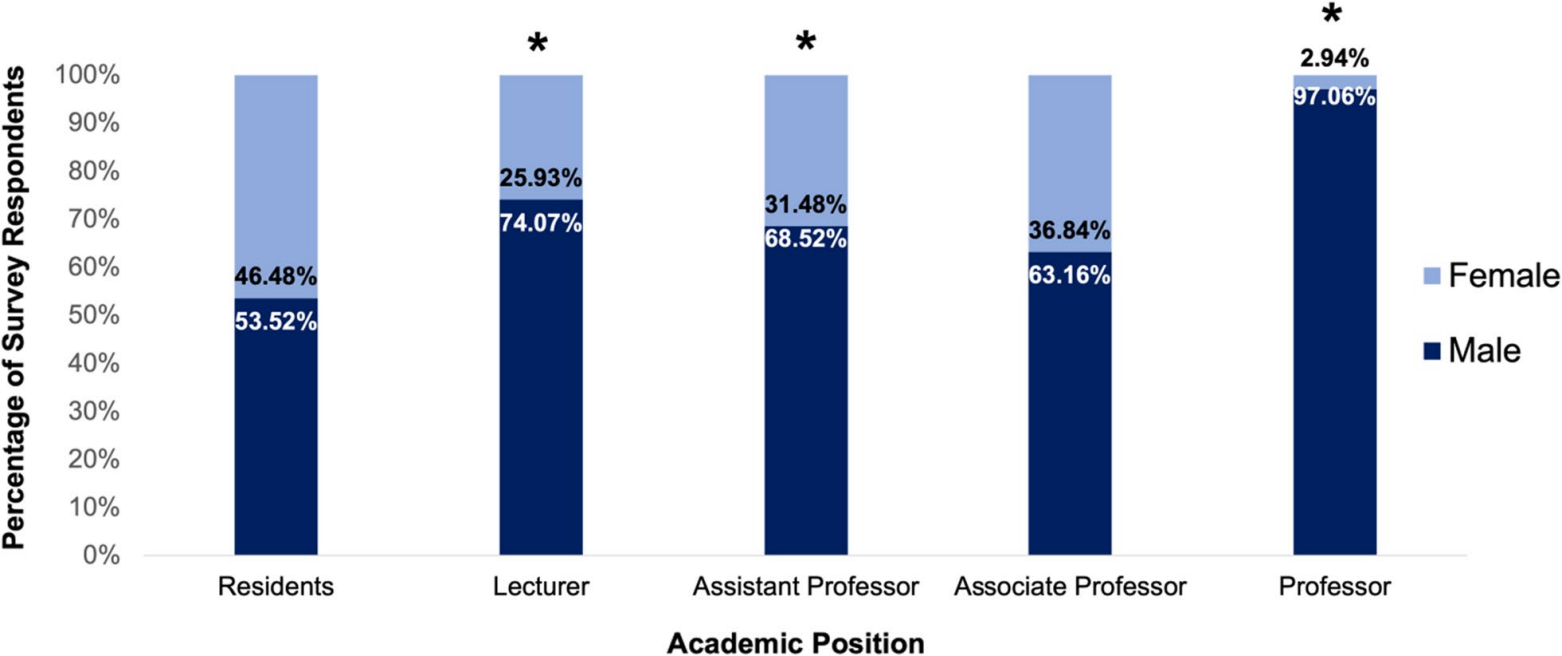
Gender distribution of survey respondents at each academic position. An "*" indicates the proportion of male and female respondents differed significantly (*p* < 0.01) from an even distribution (*n* = 224)

Of the 224 total survey respondents 71 (31.7%) were residents, 26 (11.6%) were lecturers, 54 (24.1%) were assistant professors, 39 (17.4%) were associate professors, and 34 (15.2%) were professors (Fig. 1). In total, the number of male and female survey respondents was 67.86% and 32.14% respectively, which differed significantly from an even distribution (*p* < 0.01). Of the respondents at the resident level 46.5% were female and 53.5% were male. Of the associate professor respondents 36.8% were female and 63.2% were male. The gender distribution of resident and associate professor respondents did not differ significantly from an expected even distribution (*p* = 0.55 and *p* = 0.11 respectively). Conversely the gender distribution of lecturer, assistant professor, and full professor respondents did differ significantly with 25.9% of lecturers being female and 74.1% being male (*p* = 0.01), 31.5% of assistant professors being female and 68.5% being male (*p* = 0.006), and 2.9% of professors being female and 97.1% male (*p* < 0.001) (Fig. 2.). No respondents identified as being of a gender other than male or female.

#### Racial diversity

**Fig. 3:**
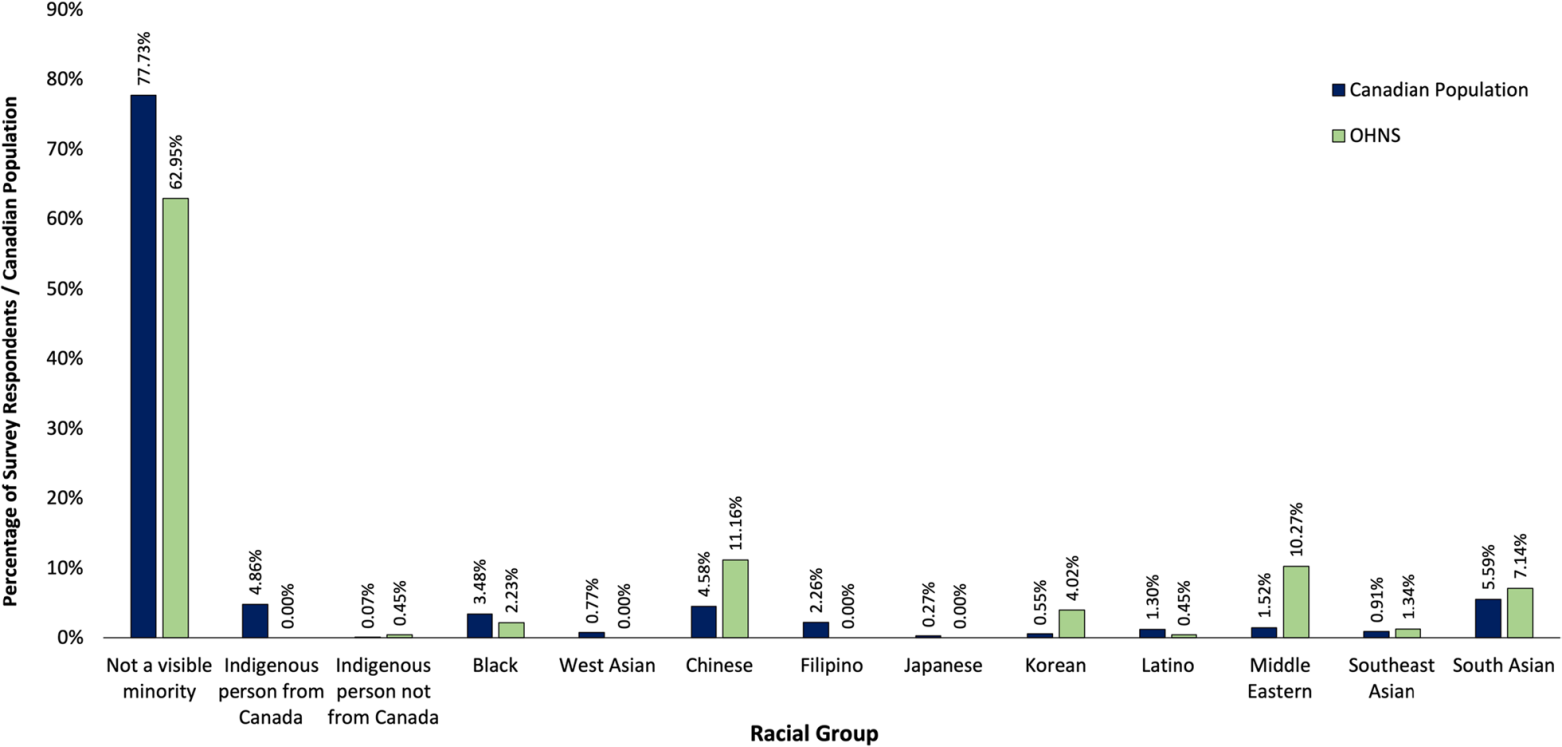
Gender distribution of survey respondents at each academic position. An "*" indicates the proportion of male and female respondents differed significantly (*p* < 0.01) from an even distribution (*n* = 224)

With regards to racial diversity, the number of survey respondents self-reported to be in in each racial group was plotted against the percentage of Canadians in these groups as reported in the 2016 Canadian census (Fig. 3). The frequency of distribution of survey respondents in each racial group differed significantly from the Canadian census (*p* < 0.01). There were 14% more individuals who identified as visible minorities in academic OHNS compared to the Canadian population. There were also a greater percentage of individuals who identified as Chinese (11.16% vs. 4.58%), middle eastern (10.27% vs. 1.52%), and Korean (4.02% vs. 0.55%) in academic OHNS programs compared to the Canadian population. No survey respondents identified as Canadian Indigenous, West Asian, Filipino, or Japanese.

**Fig. 4:**
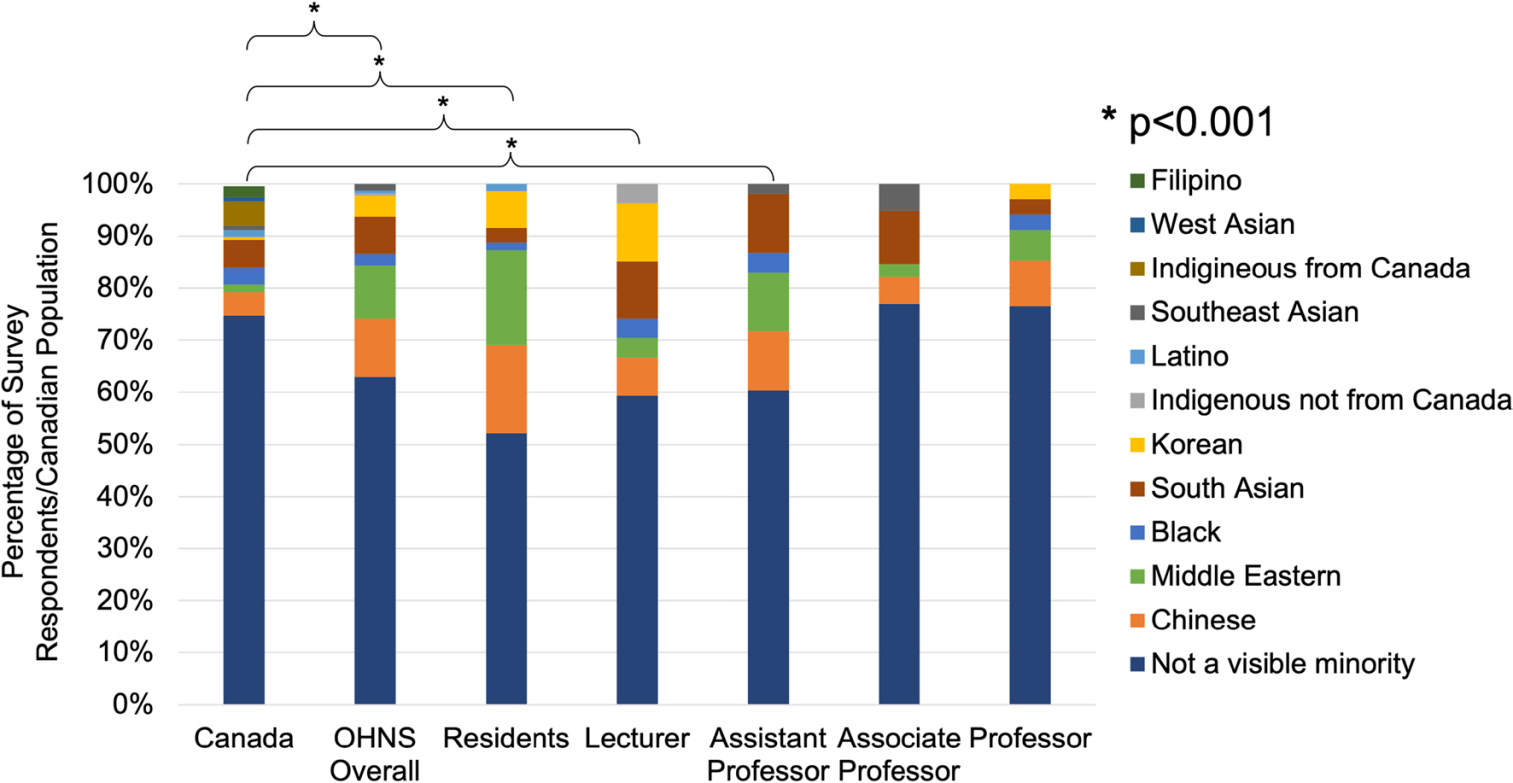
Racial demographics of the Canadian population, based on the 2016 census, plotted alongside the racial demographics of survey respondents stratified by academic position. Racial demographics of resident, lecturer, and assistant professor respondents differed significantly (*p* < 0.001) from the Canadian population (*n* = 224)

Racial demographic distribution of the Canadian population was also compared to that of the respondents’ stratified by academic position and with OHNS overall (Fig. 4.). There was a significant difference between the racial demographics of OHNS overall as well as resident, assistant professor, and lecturer respondents compared to the Canadian population (*p* < 0.001) with a lower proportion of non-minority individuals than the Canadian population. The racial demographics of the associate professor and full professor groups did not significantly differ from the Canadian population (*p* = 0.14 and *p* = 0.2 respectively).

**Fig. 5:**
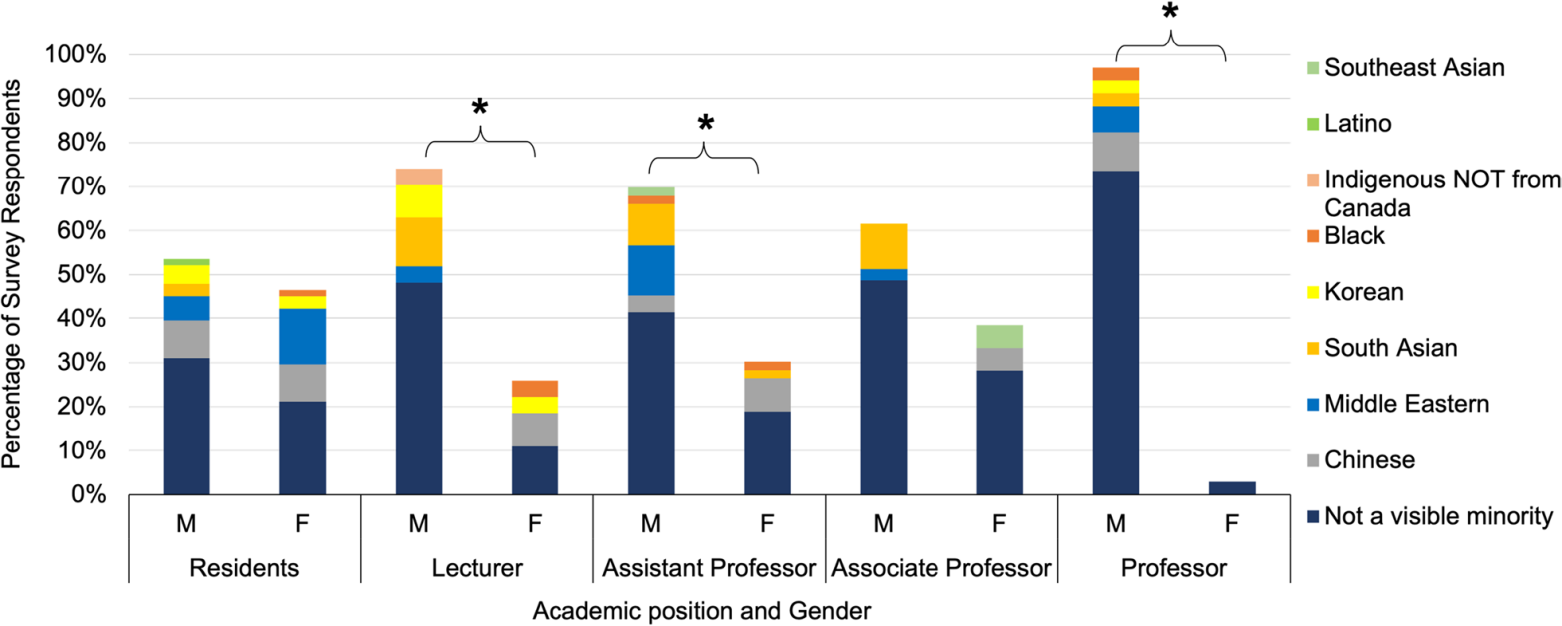
Gender and racial diversity of survey respondents stratified by academic position. There is a significant (*p* < 0.01) deviation from an even gender distribution in the lecturer, assistant professor, and professor groups (*n* = 224)

Finally, the gender and racial demographic data was assessed in conjunction with the academic group of survey respondents (Fig. 5) The significant deviation of the anticipated gender distribution in the lecturer, assistant professor, and professor groups is again seem. Additionally, there was a lack of female survey respondents from minority racial groups, most notably at the professor level with no female survey respondents being from minority racial groups.

## Discussion

The aim of this study was to quantify the landscape of race and gender diversity amongst academic OHNS programs in Canada. While gender diversity has been studied extensively, little research has been done into the racial diversity amongst academic OHNS programs in Canada. However, race and gender do not exist in isolation, and the importance of representation is well documented. For this reason, a joint analysis was warranted.

These findings illustrate that Canadian OHNS departments have a near equal ratio of male and female residents, and that there is not a significant gender disparity when looking at associate professors. However, when looking at lecturers, assistant professors, and full professors there is a significant gender disparity. This disparity is most pronounced at the professor level where only 2.9% are female. It is unclear why there was not a significant gender disparity in the associate professor group as might be expected given the significant disparity seen in the more junior positions of lecturer and assistant professor. It is possible that female faculty may be accumulating at level as they are unable to progress to full professorship, increasing the relative proportion of female associate professors. Additionally, when assessing racial demographics, OHNS residents, lecturers, and assistant professors are significantly more diverse than the Canadian population. The resident results may be somewhat skewed as 18.3% of resident respondents were Middle Eastern, likely owing to the Saudi medical trainees that are members of many resident programs. However, at more senior levels this diversity declines. This is most evident at the professor level where none of the female respondents were of a visible minority. Racial diversity greater than the Canadian population could be expected as most academic programs are in urban centers that are more culturally diverse than the Canadian population overall. For example, in Toronto 51.5% of people are visible minorities based on census data [10]. It could be anticipated that senior members of academic OHNS programs would be more diverse in keeping with the demographics of the cities they reside in. For this reason, the racial demographics of senior faculty not being significantly different than the Canadian population likely indicates reduced racial diversity compared to the communities they reside in.

The fact that men have historically outnumbered women in medicine could play a role in this disparity, however as of 1991 44.8% of medical school graduates were women and by 2020 this percentage had increased to 57% [11]. With the number of male and female medical school graduates being largely equal for decades this is unlikely to account for the gender disparity at senior positions. Looking at the gender distribution of surgical specialties specifically, data published by the Canadian Medical Association in 2019 reported 30.3% of surgical specialists were female. Comparatively 24.3% of otolaryngologists were female [12]. While there is a gender disparity in OHNS overall, our data shows the gender disparity at senior academic levels to be more pronounced.

This disparity suggests other factors are contributing to this “leaky pipeline”. Much has been said about the phenomenon of a “leaky pipeline” which describes systemic factors that filter out certain individuals leading to a lack of diversity amongst those in leadership positions. One example of this is sexism and constraints of traditional gender roles which impose the major share of family duties on women. This is just one factor that may restrict the advancement of female surgeons due to competing time pressures during and after training [13]. It may also bring some female surgeons to leave academia all together as studies have shown female surgeons to be twice as likely as their male counterparts to leave academia due to family responsibilities [14]. These systemic factors manifests in fields such as obstetrics and gynecology, pediatrics, and psychiatry where women encompass at least 50% of the specialty, but are still underrepresented in leadership positions [13]. Many additional systemic factors have also been proposed in the areas of gender bias, recruitment and training, personal factors, mentorship, and academic advancement [15]. Like other surgical specialties, many of these factors contribute to low female representation in OHNS at senior levels.

In terms of racial disparity in surgical subspecialties and OHNS, research in this area is still lacking. For many years, the diversity amongst Canadian medical students and physicians has not been documented, making it difficult to quantify demographic changes over time. In 2007, the National Physician Survey began collecting data on the ethnic and racial backgrounds of medical students and residents [16]. Available data from this survey in 2020 reported 59.0% of residents to identify as white/European [17]. While Canadian data on racial diversity is lacking, data from the United States has shown the proportion of racial minorities in OHNS to be inversely proportional to academic rank and representation of white individuals to increase with academic rank [18]. These results are consistent with our Canadian findings.

Our findings point to a lack of representation amongst several racial groups. None of the survey respondents identified as Indigenous Canadians, Filipino, West Asian, or Japanese. This lack of representation is most severe in the case of Indigenous Canadians as they represent 4.86% of the Canadian population. Indigenous children have been shown to be vulnerable to disparities in surgical outcomes [19]. A lack of representation warrants further investigation as increased indigenous representation could contribute to improved indigenous health outcomes.

Ultimately, our results indicate that at senior levels our current system continues to filter out surgeons belonging to minority racial groups. This disparity could be perpetuated by the current lack of racial diversity among faculty mentors. Poor access to minority faculty mentors could delay or impair academic advancement. Additionally, the lack of diversity amongst senior faculty and the perception of OHNS senior leadership as being nonrepresentative may deter minority individuals from pursuing academic advancement. A possible evidence-based solution to improving representation and retention of minority faculty are junior faculty development programs. One such program trialed by the University of California, San Diego School of Medicine observed faculty retention in academic medicine to increase from 75 to 90% [20]. Such programs implement a formalized mentoring process that engages senior faculty to collaborate with junior faculty on research projects, critique their work, nominate them for career-enhancing awards, promote them to chair conferences and support them to submit invited manuscripts [21]. Such programs have also been shown to be effective in promoting the advancement of female faculty [22, 23.

### Limitations

The results of this study are limited by the data collection methodology. Being a survey-based study, our results could be confounded by non-response bias. Additionally, given that the survey was distributed to members of academic OHNS programs our results do not capture otolaryngologists without academic affiliations. For this reason, our findings may not be fully representative of Canadian academic OHNS overall.

## Conclusion

The findings of this study suggest that at more junior levels gender disparity is low and racial diversity exceeds that of the Canadian population. However, at more senior levels, gender and racial diversity declines substantially. These demographic shifts are likely attributable to systemic factors such as a lack of mentorship, and traditional gender roles that affect academic OHNS, and surgical specialties in general, filtering out women and minorities. It is worth investigating what systemic barriers exist that limits the advancement of female surgeons and members of minority racial groups to senior positions to address these barriers and affect change.

## Data Availability

The datasets generated and/or analysed during the current study are not publicly available due to the possibility that participant confidentiality may be compromised. Portions of the datasets generated can be made available from the corresponding author on reasonable request.

## Abbreviations

OHNS: Otolaryngology-Head and Neck Surgery
AAMC: Association of American Medical Colleges
URM: Underrepresented in medicine
REDCap: Research Electronic Data Capture application

## References

[1] Ukatu CC, Welby Berra L, Wu Q, Franzese C (2020). The state of diversity based on race, ethnicity, and sex in otolaryngology in 2016. Laryngoscope.

[2] Branson RD, Davis K, Butler KL (2007). African Americans’ participation in clinical research: importance, barriers, and solutions. Am J Surg.

[3] Hughes TB, Varma VR, Pettigrew C, Albert MS (2017). African Americans and clinical research: evidence concerning barriers and facilitators to participation and recruitment recommendations. Gerontologist.

[4] Butler PD, Britt LD, Longaker MT (2009). Ethnic diversity remains scarce in academic plastic and reconstructive surgery. Plast Reconstr Surg.

[5] Truesdale CM, Baugh RF, Brenner MJ, Loyo M, Megwalu UC, Moore CE (2021). Prioritizing diversity in otolaryngology–head and neck surgery: starting a conversation. Otolaryngol-Head Neck Surg.

[6] Newsome H, Faucett EA, Chelius T, Flanary V (2018). Diversity in otolaryngology residency programs: a survey of otolaryngology program directors. Otolaryngol-Head Neck Surg.

[7] Johnson BC, Hayden J, Jackson J, Harley R, Harley EH (2022). Hurdles in diversifying otolaryngology: a survey of medical students. Otolaryngol-Head Neck Surg.

[8] Nieblas-Bedolla E, Williams JR, Christophers B, Kweon CY, Williams EJ, Jimenez N (2020). Trends in race/ethnicity among applicants and matriculants to US surgical specialties, 2010–2018. JAMA Netw Open.

[9] Background to CMA policy: equity and diversity in medicine 2022 [Available from: https://policybase.cma.ca/media/PolicyPDF/PD20-02S.pdf.

[10] Statistics Canada 2017. Canada [Country] and Ontario [Province] (table) Ottawa: Statistics Canada Catalogue no. 98–316-X2016001; 2017 [Available from: https://www12.statcan.gc.ca/census-recensement/2016/dp-pd/prof/index.cfm?Lang=E.

[11] Canadian medical education statistics 2020. AFMC data reports 2020 [Available from: https://www.afmc.ca/resources-data/data/reports/.

[12] Canadian medical association. Number and percent distribution of physicians by specialty and gender, Canada 2019: Canadian medical association; 2019 [Available from: https://www.cma.ca/physician-data-centre.

[13] Zhuge Y, Kaufman J, Simeone DM, Chen H, Velazquez OC (2011). Is there still a glass ceiling for women in academic surgery?. Ann Surg.

[14] Schroen AT, Brownstein MR, Sheldon GF (2004). Women in academic general surgery. Acad Med.

[15] Stephens EH, Heisler CA, Temkin SM, Miller P (2020). The current status of women in surgery: how to affect the future. JAMA Surg.

[16] Walji M (2015). Diversity in medical education: data drought and socioeconomic barriers. CMAJ.

[17] November 2020 National resident survey: summary of findings: resident doctors of Canada; 2020 [Available from: https://residentdoctors.ca/wp-content/uploads/2021/05/RDoC-Summary-of-Findings-2020-R3.pdf.

[18] Tusty M, Flores B, Victor R, Fassiotto M, Maldonado Y, Howard J (2021). The long “race” to diversity in otolaryngology. Otolaryngol-Head Neck Surg.

[19] Ingram M-C, Becker S, Olson SL, Tsai S, Sarkar A, Rothstein DH, et al. Disparities in surgical health service delivery and outcomes for indigenous children. J Pediatr Surg 2022. 10.1016/j.jpedsurg.2022.09.005 36241445

[20] Daley S, Wingard DL, Reznik V (2006). Improving the retention of underrepresented minority faculty in academic medicine. J Natl Med Assoc.

[21] Moody J. Faculty diversity: problems and solutions: Psychology Press; 2004.

[22] Laver KE, Prichard IJ, Cations M, Osenk I, Govin K, Coveney JD (2018). A systematic review of interventions to support the careers of women in academic medicine and other disciplines. BMJ Open.

[23] Fried LP, Francomano CA, MacDonald SM, Wagner EM, Stokes EJ, Carbone KM (1996). Career development for women in academic medicine: Multiple interventions in a department of medicine. JAMA.

